# Supplementary material for: Are carbon emissions trading and green financial instruments synergistic? -Comprehensive quantitative research based on content analysis
Source: PLoS One. 2024 Mar 7;19(3):e0298601. doi: 10.1371/journal.pone.0298601 (PMC10919601; doi:10.1371/journal.pone.0298601)
Supplement: S1 Data — (ZIP) [file pone.0298601.s001.zip › Data/地级市Z_F工作报告.zip!/╣π╬≈/╬α╓▌╩╨/└┤╫╘╣1⁄2╓┌║┼í╛┬φ┐╦╩2╛▌═°í┐_2007.docx]

各位代表：

现在，我代表市人民政府向大会作工作报告，请予审议，并请市政协各位委员和其他列席人员提出意见。

一、2006年工作的回顾

过去的一年，在市委的正确领导下，在市人大及其常委会的监督和市政协的支持下，市人民政府坚持以邓小平理论和“三个代表”重要思想为指导，全面落实科学发展观，认真实施“十一五”规划，紧紧依靠全市人民，抢抓机遇，开拓进取，战胜了历史上罕见的“6·8”地质灾害，全力推进工业化、城镇化和农业产业化，经济和社会各项事业全面发展，圆满完成了市十一届人大九次会议确定的各项目标任务。

（一）国民经济持续快速发展，实现“十一五”良好开局

全市实现地区生产总值270．88亿元，比上年增长14. 2％，万元地区生产总值能耗和万元规模以上工业增加值能耗分别下降1．7％和3. 2％。其中，第一产业增加值53．28亿元，增长 6％；第二产业增加值122．01亿元，增长17. 8％；第三产业增加值95．59亿元，增长15%。全社会固定资产投资121．9亿元，增长22．1％；财政收入23．08亿元，增长14. 0％；城镇居民人均可支配收入9449元，增长16．4％；农民人均纯收入2879元，增长11．8％。全市经济在多年保持两位数增长的情况下仍然高于10％，完成情况比预期好，比前几年好，经济增长的持续性和稳定性明显增强。

（二）工业建设大会战成效显著，工业经济不断壮大

在工业建设大会战123个项目中，实际开工项目117个，完成计划投资目标的项目90个，共完成投资30. 55亿元，占年度投资计划的81．9％。规模以上工业分别完成总产值177．15亿元、增加值58．55亿元，分别增长36．1％和32．5％；盈亏相抵后实现利润4. 77亿元，增长35．0％；实现税收6．23亿元，增长27. 9％。工业对经济增长的贡献率达52．7％，拉动GDP增长7．6个百分点。规模以上工业完成新产品产值6．42亿元，增长206％。全市亿元产值工业企业达45家，比上年增加13家。县域规模以上工业完成增加值29．85亿元，对全市规模以上工业增长贡献率达58％。梧州工业园区完成固定资产投资3．5亿元，完成工业增加值8000万元。

（三）农业和农村经济稳步发展，新农村建设试点成效明显

全市农林牧渔业总产值达91．16亿元，增长6. 03％；粮食种植面积244. 4万亩，总产量91万吨，其中优质稻面积168．1万亩，种植比例达88％。农业“五大措施”的作用进一步显现，新增农产品基地面积17．59万亩，实现农产品销售4．04亿元；培训农民工50281人，推荐就业51168人；实施农村基础设施项目168项，完成投资1．56亿元。在实施“五大措施”的基础上，我们选出条件和基础较好的27个村作为新农村建设试点，投入资金1亿多元，基本完成试点村的道路、水毁民房、供水供电设施、文体卫生设施、村容亮化等主要建设项目，初步实现了“五通”、“五化”、“六有”的目标。

（四）城市基础设施建设取得新进展，城镇化进程不断加快

我们开展了以建设十五大标志性建筑为重点，以打造岭南山水名城为目标的新一轮城市建设。城市基础设施完成投资26．9亿元。宝石大厦、星朗名都、中银大厦、汇景国际公寓等十五大标志性建筑完成年度建设任务。新增城市道路2. 5公里、排水渠3．5公里，新增绿化面积3万平方米，新增路灯2000盏。市容市貌进一步美化、亮化。积极实施“城乡清洁工程”，城市管理逐步走向成熟，城乡文明程度进一步提高。各县（市）县城和集镇建设力度加大。城镇建设完成投资6．84亿元，人均住宅建筑面积达28平方米。全市城镇化水平达32．6％。

（五）固定资产投资力度加大，重大项目建设扎实推进

全年建设项目503个，其中新开工项目352个，投产项目154个，当年固定资产投资对经济增长的贡献率达35％。长洲水利枢纽完成投资13亿元，洛湛铁路梧州段、南梧高速公路苍梧至岑溪段分别完成投资12亿元、4. 5亿元。桂梧高速公路马江至梧州段已全线开展征地拆迁等工作，完成投资1亿元。市区地质灾害治理第一批政府投资项目完成投资1094. 97万元。旺村水利枢纽、西江航道梧州段整治已开工建设。一批重大项目前期工作取得新突破，林浆纸一体化项目正式启动，项目原材料基地建设工作全面开展；年产20万吨木薯燃料乙醇项目已完成项目初步选址和原料基地规划工作，招商工作正在抓紧进行；桂东火电项目和核电项目已完成可研和预评审工作；梧州新港赤水圩作业区码头前期工作全面推进，争取今年开工建设。

（六）招商引资成效显著，对外开放进一步扩大

全市招商引资实际到位资金84．6亿元，增长27．6％。其中，实际利用境外资金1．29亿美元，增长13％；引进市外境内资金74. 3亿元，增长30. 8％。在新引进的338个项目中，工业项目205个，占引进项目总数的56．3％；东部产业转移项目193个，合同投资额57. 2亿元。成功举办第三届梧州国际宝石节，落实招商引资项目61个，投资总额20．93亿元，贸易成交额3．58亿元。狠抓外贸出口工作，出口市场不断扩大，出口商品结构进一步改善，重点产品出口稳步增长。全市实现外贸出口总额2．5亿美元，增长1．6％。

（七）各项事业全面进步，社会保持和谐稳定

人民生活得到改善。城镇居民人均可支配收入、农民人均纯收入均有较大幅度增长。城乡市场兴旺，实现社会消费品零售额98．04亿元，增长14. 6％。

就业再就业工作取得新成效。全年新增城镇就业1．5万人，占计划的115．4％。下岗失业人员实现再就业6000人，占计划的109．1％；帮扶大龄就业困难人员实现再就业2200人。城镇登记失业率为4．05％，比控制数4. 5％低0．45个百分点。社会保障体系进一步完善，城镇社会保险覆盖面不断扩大，养老、失业、工伤保险参保人数进一步增加，农民工参保逐步铺开。城镇居民最低生活保障继续巩固。农村最低生活保障全面启动。

科技、教育、卫生、文化、广播电视等事业有较大发展。科技创新体系建设加快推进，自主创新能力有新提高。农村义务教育得到加强。全面完成农村中小学危房改造“三年计划”。梧州学院正式挂牌成立。公共卫生、基本医疗服务体系、重大疾病防控体系建设取得新进展。文化建设全面推进，文化产业加快发展。全面完成有线电视数字化整体转换工作，我市是全国最早开通数字电视的5个城市之一。精神文明建设不断加强，干部群众思想道德水平和文明素质有了新的提高。

“平安梧州”建设取得显著成绩。加强和完善基层治安防范体系，强化了派出所、司法所、法庭等政法基层基础建设，建立起较为完善的群防群治组织管理体制和工作机制，社会治安综合治理各项措施得到全面落实。

为民办实事项目全面完成。自治区政府确定为民办的8件实事中，与我市有关的已全部落实，市政府承诺为民办的10件实事已顺利完成，分别是地质灾害高危地段住户搬迁、水上居民搬迁、建设新兴二路小学、全市农村义务教育阶段中小学免收学杂费、农村中小学危房改造、新建或扩建岑溪市14个乡镇卫生院、新建或改建乡村公路15条、新建或改造水利工程18项、帮助4300名下岗失业人员再就业、新建经济适用房3．5万平方米。

各项事业全面发展。人口和计划生育工作强化一把手责任，严格实行目标管理责任制，取得明显进步。资源合理开发利用，环境保护和生态建设得到加强。人才强市战略深入实施，人才队伍进一步发展壮大。工会、青少年、妇女儿童、老龄、残疾人等各项事业稳步发展。政府职能部门各项工作取得了新的成绩。中直、区直驻梧单位为我市经济社会发展提供了有力保障，作出了积极贡献。驻梧部队为我市经济社会发展特别是在抗灾救灾、突发事件处置等方面发挥了重要作用。全市呈现出经济加快发展、社会和谐稳定的良好局面。

（八）政府自身建设得到加强，发展环境进一步改善

自觉接受人大监督和政协民主监督，广泛听取社会各方面的意见和建议。人大代表议案、建议，政协委员提案和社会各界来信来访的办理水平进一步提高。市本级办理市人大代表议案、建议49件，市政协委员提案140件，按期办复率达100％。深入开展“两整一会战”等活动，公务员队伍建设进一步加强，机关作风明显好转，投资环境明显改善。切实加强督查工作，加大各项政策措施落实情况的督查力度，确保政令畅通，创造良好的政务环境。加快政府职能转变，行政管理体制改革进一步深化，积极推进政企分开、政务公开和电子政务，政府经济调节、市场监管、社会管理和公共服务职能进一步增强。认真实施《全面推进依法行政实施纲要》，政府机关依法行政水平和工作效率不断提高，行政审批制度改革不断深化。加强政风行风建设，深入开展反腐败斗争，切实纠正损害群众利益的不正之风，严肃查处了一批违法违纪案件，解决了群众反映比较强烈的问题。

回顾过去的一年，我们能够圆满完成经济社会发展各项任务，主要是在具体工作中注重把握以下几个方面：

第一，在加快发展中，我们紧紧咬住经济建设不放松，努力做到科学发展。我们竭尽全力加快发展步伐，壮大经济总量。所有工作的部署、举措、落实和问题的解决都围绕经济建设这个中心，从而推动了经济社会进入科学发展轨道。

第二，在加快发展中，我们突出工作重点，强化责任，行必责实。我们狠抓工业总量壮大、城市改造、项目投资、招商引资、宝石节举办、为民办实事等重大工作。在工作中，注重落实责任，对重大工作制定具体组织实施方案，加强督查落实，强化激励措施，充分调动各方面的积极性。

第三，在加快发展中，我们注重落实工作措施，破解难题。我们针对工业和投资环境的薄弱环节，开展了“两整一会战”；针对企业发展难、税源缺的情况，组织了企业服务队，帮助企业解决困难，增加财税收入；针对招商引资存在的问题，制定了一系列优惠政策和奖励措施，吸引客商来梧投资。这些措施的深入实施，使各项工作顺利推进，成效明显。

第四，在加快发展中，我们始终目标方向明确、工作思路清晰。根据我市实际，我们重点研究制定了工业化、城镇化、支柱产业发展、东部产业转移等专项规划并提出了具体发展思路和工作重点，使我市今后五年的发展布局得到了完善和深化。

第五，在加快发展中，我们高度关注民生，促进社会和谐稳定。切实加强了就业、社会保障、救灾救济和为民办实事工作，着力解决涉及人民群众切身利益的问题，让广大群众共享改革发展成果。

过去的一年，我市经济社会发展取得了可喜的成就。这得益于自治区党委、政府和市委的正确领导，得益于市人大、市政协的监督与支持，是全市上下真抓实干、艰苦奋斗的结果。在此，我代表市人民政府，向全市人民，各位人大代表、政协委员，各民主党派、各人民团体和社会各界人士，驻梧部队官兵，以及所有关心支持我市改革开放和现代化建设事业的各界朋友，致以崇高的敬意和衷心的感谢！

各位代表，对照全市人民的期望，对比区内同级城市的发展，我市经济社会发展还有一定的差距，仍然存在不少困难和问题：一是经济总量不大，质量不高。二是工业化、城镇化水平还比较低。三是财政收入总量偏小，非税收入比例过高，财政收支矛盾突出。四是县域经济发展还有差距，农业基础薄弱，农业产业化水平较低。五是社会保障体系仍待完善，就业和再就业压力较大，低收入群体生活水平有待提高等。对于这些困难和问题，我们将高度重视，采取有效措施加以解决。

二、2007年政府工作的总体要求和主要任务

2007年是本届政府施政的第一年，也是我市全面落实科学发展观，深入实施“十一五”规划，加快建设富裕文明和谐新梧州的重要一年。根据市第十一次党代会精神，今年政府工作的总体要求是：坚持以科学发展观为统领，继续实施开放带动和项目带动两大战略，加快融入珠三角步伐，狠抓工业化、城镇化和农业产业化，努力实现经济又好又快发展。据此，今年我市经济社会发展的主要目标为：地区生产总值增长13％，力争增长15％；万元地区生产总值能耗和万元规模以上工业增加值能耗分别下降1．7％和3．2％以上；全社会固定资产投资增长20％，力争增长22％；财政收入增长12％，力争增长14％；规模以上工业增加值增长25％，力争增长30％；外贸出口总额增长13％；实际利用外资增长6％；引进市外境内资金增长10％；社会消费品零售总额增长12％；城镇居民人均可支配收入增长8％；农民人均纯收入增长8％；城镇新增就业1．5万人，城镇登记失业率控制在4．5％以内；人口自然增长率控制在11‰。以内。

上述预期目标，是综合分析全区形势，结合我市实际，本着既积极可行又留有余地的原则提出来的。在我市经济连续多年高速增长，制约因素和不可预见因素较多的情况下，我们实事求是地提出上述预期目标，有利于为深化改革、加快发展提供较为宽松的环境，有利于为产业结构调整创造更大的空间，有利于我市经济社会全面、协调和可持续发展。

为实现上述目标，今年政府工作要把握好以下几个关键问题：

一是抢抓东部产业转移难得的发展机遇。今后几年是东部产业转移的黄金时期。自治区对我市的定位是打造成为承接东部产业转移的重要基地。我们能否把东部产业吸纳过来，能否在全区成为承接东部产业转移的“领头雁”，对我市今后经济又好又快发展至关重要。机遇难得，稍纵即逝。我们必须树立强烈的机遇意识、危机意识和责任意识，增强抓住机遇的紧迫感，按照既定的招商引资方向和目标，狠抓各项措施落实，力争今年承接东部产业转移有重大突破。

二是着力解决制约经济发展的“瓶颈”问题。着力解决项目建设用地偏紧、交通不发达、项目资金筹措难等问题。严格执行土地利用总体规划和年度用地计划，统筹安排，节约集约用地，确保重大项目建设用地需求。着力推进公路、铁路、水路、航空等交通发展，形成便捷、通畅、高效、安全的现代综合交通网络，以发达的交通促进产业转移。采取多种形式，扩大贷款规模，吸纳集聚民间和区外、境外资本，多渠道筹措资金，加大投资担保力度，解决我市融资难的问题。

三是狠抓重大项目的落实，壮大经济总量。重大项目建设是我市提速发展的重要因素。实践证明，没有重大项目的发展，就没有梧州经济的腾飞。今年要作为重大项目建设年、推进年。按照一个项目、一个领导挂帅、一个部门为主负责、一个工作班子跟踪服务的“四个一”要求，加大工作力度，全面推进重大项目建设，确保再上1至2个重大项目。

四是进一步优化发展环境。继续开展“两整一会战”活动，完善机关作风考评制度，进一步改善服务环境，真正把审批办证大厅建设成为高效率的服务窗口，切实为外商提供一流质量、一流效率的服务。推动全市机关作风明显改善，服务意识明显增强，工作效率明显提高，投资环境明显优化。

五是把造福群众作为政府工作的不懈追求。坚持以人为本、民生为主的原则，树立正确的政绩观，始终坚持把群众利益放在首位，把群众冷暖挂在心上。从人民群众最关心、最直接、最现实的利益问题出发，协调处理好各方面的利益关系，多为群众办实事、办好事，让群众得实惠。

具体来说，要抓好以下几个方面工作：

（一）突出抓好工业发展，加快工业化进程

推进重大工业项目建设。重点抓好林浆纸一体化、20万吨木薯燃料乙醇、梦都美地毯等15个重大工业项目，加快列入会战的工业项目建设进度，进一步加大招商引资力度，争取引进一批新的重大工业项目。

推进现有企业做大做强。着重抓好具有比较优势、产业带动作用大的企业，培育形成一批年销售收入超10亿元、超5亿元或超亿元的企业，重点培育30家优势企业。通过做大做强企业，加快优势产业发展，打造我市十大工业支柱产业。

大力承接东部产业转移。做好产业转移的总体布局，重点推进产业集中区建设。采取“工业飞地”的办法，由市和县（市、区）两级共同招商，按产业规划、利税分享的办法共同建设集中区。研究制定集中区的财政贴息、信贷支持等优惠政策鼓励投资，引进同类产业或产业上下游企业形成产业集群。

加快工业园区建设步伐。按照“一园多基地”布局的要求，优化工业园区或工业集中区的项目布局，加快基础设施建设，积极推进已入园项目加快建设投产；加快土地清理，争取引进一批新的项目。今年，梧州工业园区要完成固定资产投资5亿元，税收比上年有较大幅度增长。同时，积极推进藤县赤水临港工业区的前期工作，尽快启动园区建设。

（二）突出抓好现代农业发展，加快推进新农村建设

努力抓好现代农业。把发展现代农业作为新农村建设的首要任务，在确保粮食总产量不减少的基础上，大力发展特色优势产业，着力培育亚热带名优水果、木薯、桑蚕、六堡茶、中草药、八角香料、速丰林、三黄鸡、瘦肉型猪、江河网箱名优鱼类等十大农业优势产业。大力培育农业龙头企业，加快建立农业优势产业招商引资项目库，吸引国内外资金兴办龙头企业。继续重点扶持15至20家农产品加工企业，支持企业建立原材料生产基地。通过建立相关利益机制，带动农业产业化发展，促进农产品转化增值和农民增收。

加强农村基础设施建设。加强农村交通建设，计划新开工通乡油路4条，总投资6315万元；通村油路50条，总投资3800万元；农村通达工程10项，总投资1100万元。加强农村水利、生态能源等建设。继续抓好长洲防洪堤正阳堤段及藤县防洪堤工程建设。加强农村水毁水利工程建设，实施14处农村饮水解困工程，帮助4.19万人解决饮水困难问题。加大农机装备投入，提高农业机械化综合水平。新建农村沼气池7000座，并配套“三改”及服务网点建设。

推进新农村建设试点工作。按照社会主义新农村建设要求，加强规划指导工作。在巩固去年试点工作成效的基础上，进一步扩大试点范围，以点带面，推进新农村建设不断取得进步。

（三）突出抓好交通基础设施建设，加快构建立体交通体系

公路方面：重点推进广梧、桂梧、南梧高速公路建设，推进岑溪至筋竹、梧州至平南高速公路建设以及梧州至肇庆一级路改造。继续推进乡村公路建设大会战，加快藤县潭东至太平、藤县至容县等路网工程建设步伐。

铁路方面：加快推进洛湛铁路梧州段建设，全力支持推进南宁至广州城际快速铁路建设，配合做好岑溪至罗定铁路建设。

航道码头方面：加快推进西江航道疏浚工程建设和梧州新港赤水圩作业区码头、李家庄码头二期、三期工程以及苍梧龙圩深水码头建设，加快河西码头搬迁步伐。

机场建设方面：积极推进与有关单位的合作洽谈，争取今年恢复梧州机场航班。

（四）突出抓好中心城市建设，加快城镇化进程

拓展城市发展空间。加快实施红岭新区修建性详细规划，抓好新区基础设施建设，加快推进西江三桥、红岭路网、火车站站前大道、快速干道建设，争取红岭新区建设在今年能够有较大进展。做好苍梧片区的规划调控，增强中心城市辐射带动能力。

加快城市标志性工程建设。着重推进宝石大厦、蓝天港湾、汇景公寓和西江明珠塔等十五大城市标志性建筑的建设进度，完善配套设施，并努力增加一批新的标志性建筑。努力推进西堤路沿线开发建设，把西堤路建设成为城市的新亮点。

继续开展城市改造。重点抓好蛇园改造、华杰商贸城以及白垢等部分旧城及城中村改造工作。积极推进城区工业企业尽快“退城进郊”。大力实施一批城建“民心工程”，加快改造大学路和蝶山一、二路，改造石鼓路和桂江一、二路沿街建筑立面，整治河西部分内涝点。进一步改善城市环境，树立我市作为广西东大门的新形象。

大力推进“城乡清洁工程”。建立“城乡清洁工程”的长效管理机制，全面开展城乡市容环境综合整治工作，严管重罚，全面治理“五乱”等行为。坚决拆除违规违章建筑，重点整治市区东、南、北面3个出口沿线的环境。加强对城市超标排放废水、废气、噪音等违法行为的监督管理和整治，提高城市环境质量整体水平。

（五）突出抓好旅游商贸业，加快第三产业发展

打造旅游精品。市区重点抓好“一山一寺一塔一庙”旅游精品建设，加快推进白云山景区改造、四恩寺配套设施建设、西江明珠塔建设、骑楼城创国家4A级景区、龙母太庙扩建等项目建设。继续支持岑溪吉太漂流和石庙景区、苍梧县飞龙湖景区和李济深故居、藤县太平狮山和石表山景区、埌南黎寨蝴蝶谷、蒙山太平天国封王建制遗址等开发建设。加强旅游区域合作，组建旅游营销联盟，与肇庆、贺州等地共同打造“广肇梧”黄金旅游线路和桂东宗教旅游精品线路。改善旅游接待服务设施，促进部分酒店上星级、上档次，提高旅游接待能力和服务水平。

加强现代市场体系建设。大力发展和培育现代物流业，鼓励国内外物流企业参与我市物流设施建设和经营。加快同舟农产品物流配送中心、福兴时代广场等商贸设施建设。努力引进客商建设一批专业市场。大力实施“万村千乡市场工程”，全面推进农村生活消费品、生产资料和农产品流通网络建设。整顿和规范市场经济秩序，加快商业领域信用体系建设，营造良好的消费环境。

（六）突出抓好招商引资，加快融入珠三角步伐

落实招商引资责任制。继续实行招商引资激励机制和重大项目协调服务机制，提高招商引资成效。抓好签约项目的跟踪落实，建立招商引资档案及定期通报工作制度。促进签约项目快落实、快上马，提高合同履约率、项目开工率和资金到位率。

突出招商引资重点。在区域上，以粤港澳为招商引资的重点区域，加大对珠三角区域招商力度，拓展长三角，发展日韩和东盟地区。在产业上，围绕我市优势产业，包装策划一批有吸引力的精品项目。着重抓好木薯燃料乙醇等大型工业项目，抓好玩具业、纺织业和以宝石深加工带动的饰品产业，抓好以果蔬食品深加工带动的开发性农业等，打造资源型产业集群。

创新招商引资方式。实行驻点招商和组织小分队招商。加强与珠三角企业和招商协会的联系，有针对性地举办招商推介活动。派出小分队分别到日本、韩国和新加坡、港澳举行项目推介，吸引更多客商到我市投资。继续办好第四届梧州国际宝石节，不断提高举办宝石节的成效，扩大其影响力。

优化招商引资环境。进一步优化投资软环境，依法维护外商合法权益。建立健全外来投资项目联合审批制度和项目建设工程设计图纸联合审查制度。对重大项目实行联席审批，简化审批程序，减少审批环节，提高办事效率。进一步规范中介机构服务性收费，鼓励金融机构对重点项目和有潜力的项目给予资金支持。规范县（市、区）土地优惠政策和产业布局政策。

（七）突出抓好民生工程，加快和谐社会建设步伐

着力解决民生问题。将就业再就业列入各级政府责任目标，优先解决“4050”人员和城镇“零就业家庭”的就业问题，实现城镇家庭户户有人就业。全市新增城镇就业人数要达1．5万人，农村劳动力转移就业5．14万人；失业保险参保人数10．5万人。继续做好养老保险基金征收工作，进一步扩大医保覆盖面，加强和规范城镇低保工作，全面推进农村困难群众最低生活保障，建立健全城乡社会救助体系。贯彻落实最低工资制度，切实维护外来务工人员的权益。积极推进贫困村的扶贫开发，促进贫困村农民脱贫致富。全力办好自治区政府今年确定的十件为民办实事项目。在此基础上，把地质灾害整治、水上居民搬迁、办理农房保险60万户作为我市今年为民办实事的项目，坚决完成计划任务。

促进社会事业加快发展。实施第三轮科技创新计划，提高企业科技创新能力，加强县域科技服务，促进农村科技进步。继续深化人事制度改革，加强以公务员和专业技术人员为重点的人才队伍建设。抓好基础教育，建立健全农村中小学教育经费保障新体制，全面完成“两基”攻坚任务，调整优化中小学布局，加快市十五中和新兴二路小学等项目建设；建立有效的教育资助体系，绝不让一个学生因家庭贫困而失学；大力发展职业教育，启动市职业技术学院建设，争取打出具有特色的职校品牌；加强梧州学院基础设施建设，提高教学质量。加强医疗卫生工作，加大社区卫生服务建设力度，加强疾病预防控制和妇幼保健工作，加强食品卫生、药品安全的监督管理。推进新型农村合作医疗建设，加快建设覆盖城乡居民的基本卫生保健制度，为群众提供安全、方便、价廉的公共卫生和基本医疗服务。努力构建公共文化服务体系，推动公益性文化事业发展，加强基层文化设施建设，抓好县级文化馆、图书馆建设；继续实施文化精品战略，大力发展文化产业；强化文化市场管理，营造健康向上的社会文化环境。加强城乡社区体育设施建设，力争开工建设市体育中心、青少年活动中心和妇女儿童服务中心；推动全民健身运动深入开展，提高竞技体育水平。加强民兵预备役建设，以开展争创全国、全区双拥模范城为契机，继续推进双拥工作深入开展。

加强人口、资源和环境工作。稳定低生育水平，降低政策外生育，提高人口素质，综合治理出生人口性别比偏高问题。采取有效措施，坚决扭转人口和计划生育工作被动、落后的局面，将人口自然增长率控制在11‰以内。加强资源节约和环境保护，加快建设资源节约型、环境友好型社会。突出抓好重点行业节能减排，抓好水污染和大气污染防治，严格执行节能减排目标责任制。

加强社会治安综合治理。健全社会治安防控体系，坚持打、防、控、管相结合，依法严厉打击各种刑事犯罪，维护社会稳定，切实保障人民群众生命财产安全。完善信访工作责任制和社会纠纷调处机制，努力做到人民内部矛盾早发现、早介入、早解决，把矛盾解决在基层和萌芽状态。建立健全各种自然灾害和公共安全突发事件监测预警体系和信息报告、应急救援、社会动员机制。高度重视安全生产，努力构建平安梧州。

（八）突出抓好增收节支，加快构建公共财政

促进财税增收。培育财源和强化税收征管并举，确保财政稳定增长。把开源的重点放在工业上，把挖潜的重点放在第三产业上。支持一批重点财源项目的建设和发展，加快税源建设，积极跟踪落实项目进度。完善招商引资项目考核管理办法，促进项目尽快投产，尽快产生税收。加快建立以产业为重点的“财源建设带”，稳定支柱财源，扩大中小税源，实现年纳税10万元以上的企业达到1300家。进一步推进财源建设目标考核管理制度，切实做好纳税10万元以上的基础财源企业的跟踪服务工作，重点跟踪30户重点企业税收完成情况。加强契税、耕地占用税直征工作，努力挖掘增收潜力，确保应收尽收。

理顺分配关系。进一步调整和完善市区财政管理体制，切实解决城区建筑、房地产开发项目税收混库和新办企业税收分成等问题，调动城区组织税收的积极性。全面推进部门综合预算管理制度改革，规范收入分配制度，改变部门间“苦乐不均”的现状。加强非税收入管理，所有行政事业性收费全部纳入财政预算管理。继续深化完善财政投资评审、政府采购、国库集中支付等改革，推进财政资金绩效评价，提高资金使用效益。

集中财力办大事。财政资金全力支持我市工业建设大会战，支持企业做大做强。积极筹集新农村建设资金，支持农业基础设施建设，提高农业抗灾防灾能力。进一步落实“两免一补”、就业和社会保障等各项民心工程，切实保证工资发放、党政机关正常运转和各项法定支出。对于具有相同性质、相同使用方向的各项专项支出，要整合使用，形成资金合力，充分发挥财政资金的引导、带动和放大作用。

（九）突出抓好政府自身建设，加快建设效能政府

加强三项制度建设。加强以行政效能建设为核心的行政效能、政府执行力和政务环境建设。建立和健全首问负责制，全面推行限时办结制，严格实行责任追究制等三项制度，确保工作作风明显改善，办事效率明显提高。

积极为群众解决实际问题。大力弘扬清简务本、行必责实的工作作风，坚持以人为本，从人民群众的根本利益出发，深入基层调查研究，了解广大人民群众的疾苦，关心和关注群众的生活、生产等问题。努力解决影响改革发展稳定的突出问题，切实为基层、为群众排忧解难。

推进民主法制进程。加强公务员队伍建设，提高公务员队伍素质。强化行政执法监督，规范行政执法行为。进一步推进民主法制建设，加快建立和完善重大问题集体决策制度、专家咨询制度、社会公示和社会听证制度。

抓好反腐倡廉工作。始终牢记“两个务必”，大力发扬艰苦奋斗和勤俭节约的精神。坚决反对铺张浪费、拜金主义、享乐主义和奢靡之风。加快建立健全教育、制度、监督并重的惩治和预防腐败体系，深入开展治理商业贿赂专项工作，严肃查处以权谋私、权钱交易等腐败案件，坚决遏制腐败现象发生。

各位代表，新的一年我们面临着前所未有的新机遇，也面临着前所未有的新挑战。我们相信，在市委的坚强领导下，在市人大的监督和市政协的支持下，只要全市人民团结一致，振奋精神，开拓进取，扎实工作，就一定能够完成今年各项工作任务，以加快建设富裕文明和谐新梧州的优异成绩迎接党的十七大胜利召开！
